# Perception of Community Pharmacists towards Dispensing Errors in Community Pharmacy Setting in Gondar Town, Northwest Ethiopia

**DOI:** 10.1155/2017/2137981

**Published:** 2017-05-22

**Authors:** Dessalegn Asmelashe Gelayee, Gashaw Binega Mekonnen

**Affiliations:** ^1^Department of Pharmacology, College of Medicine and Health Sciences, University of Gondar, Gondar, Ethiopia; ^2^Department of Clinical Pharmacy, College of Medicine and Health Sciences, University of Gondar, Gondar, Ethiopia

## Abstract

**Background:**

Dispensing errors are inevitable occurrences in community pharmacies across the world.

**Objective:**

This study aimed to identify the community pharmacists' perception towards dispensing errors in the community pharmacies in Gondar town, Northwest Ethiopia.

**Methods:**

A cross-sectional study was conducted among 47 community pharmacists selected through convenience sampling. Data were analyzed using SPSS version 20. Descriptive statistics, Mann–Whitney* U* test, and Pearson's Chi-square test of independence were conducted with *P* ≤ 0.05 considered statistically significant.

**Result:**

The majority of respondents were in the 23–28-year age group (*N* = 26, 55.3%) and with at least B.Pharm degree (*N* = 25, 53.2%). Poor prescription handwriting and similar/confusing names were perceived to be the main contributing factors while all the strategies and types of dispensing errors were highly acknowledged by the respondents. Group differences (*P* < 0.05) in opinions were largely due to educational level and age.

**Conclusion:**

Dispensing errors were associated with prescribing quality and design of dispensary as well as dispensing procedures. Opinion differences relate to age and educational status of the respondents.

## 1. Introduction

One of the key functions of pharmaceutical care is dispensing medications, which involves selecting medications, transferring them to a container, and product labeling. Community pharmacies are often the first point of contact in the healthcare system because of easy accessibility and smooth approaches to patients [1]. These settings therefore have an important role in promoting rational drug use. According to the World Health Organization (WHO), rational use of drugs refers to situations when the right medicines are given to patients in appropriate doses and for an adequate period of time at the lowest cost to them and their community [2]. The International Pharmaceutical Federation (FIP) emphasizes that pharmacists are responsible for offering the required information for the quality use of medications [3]. However, comprehensive drug information has not been usually given to patients. Medicine's name, indications, dosage, and directions for use are more commonly communicated to patients compared to drug interactions, side effects, contraindications, precautions, and storage conditions [4]. Thus, proper training of drug dispensers to effectively communicate such aspects of drugs has been suggested [5].

Despite being cornerstones of therapy in healthcare, medications remained to be common sources of error and harms. Medication error (ME) refers to preventable events such as those related to prescribing, dispensing, and use which contribute to inappropriate use of medications and patient harm [6]. The most prevalent type of medication error is dispensing error and it refers to deviations from the prescription order regarding type, dose, storage, and so forth of drugs [7]. Such errors are a significant cause of preventable adverse events. The rates of dispensing errors were reported to be 0–45% across different studies [8]. In developing countries, the main types of dispensing errors include the supply of wrong drugs and wrong directions [9]. Several causes of dispensing errors have been reported including high workload, similar drug names, similar drug packaging, staffing levels, interruptions, and poor handwriting [9].

The dispensing errors are important targets for patient safety interventions [10]. However, there are few studies regarding medication errors in Ethiopia [11–13]. And to the best of our literature search, data regarding the perception of pharmacists towards dispensing errors are scarce. Therefore, this baseline study is intended to assess the perception of community pharmacists working in Gondar town, Northwest Ethiopia, towards the factors contributing to and perceived solution on dispensing errors in the community pharmacies.

## 2. Methods

A cross-sectional study was conducted among pharmacists working in the community pharmacies in Gondar town, Northwest Ethiopia, from October to December 2016. As of 2014, the town has one referral and teaching hospital, one private general hospital, a number of health centers and private clinics, and 53 medication retail outlets (19 pharmacies and 34 drug stores). In this study, community pharmacist refers to at least diploma holders in pharmacy education and community pharmacy refers to both drug stores and pharmacies.

The data collection instrument was a structured self-administered questionnaire adopted from a previous study by Peterson et al. [14] with some modifications. It consisted of closed questions of yes/no type and 5-point Likert-type scale questions (never, rarely, sometimes, often, and very often) on sociodemographic characteristics, frequency of risks and actual dispensing errors, and perceived factors contributing to and strategies to minimize dispensing errors as well as common type of dispensing errors happening in community pharmacies.

The questionnaire was pretested on 5 pharmacy technicians working part-time in private pharmacies. Necessary modifications were made before distributing the questionnaires in person to the pharmacies. All consenting pharmacists (*N* = 47) working in the community pharmacies were involved in the study. The collected data were cleared and entered into the computer and analyzed by using the Statistical Package for Social Sciences (SPSS) version 20.0 for windows (SPSS Inc., Chicago, Illinois). Though the questionnaire was validated in the previous study, the modified one used in our study was also tested for its reliability. The reliability of the different subcomponents of the questionnaire was measured and Cronbach's alpha value was 0.821 (perceived factors contributing to dispensing errors, 11 items), 0.905 (perceived solutions to minimize dispensing errors, 10 items), and 0.856 (type of common dispensing errors, 4 items).

The results were described in terms of frequencies, percentages, and means ± standard deviations. The relationships among variables were analyzed by using Mann–Whitney *U* test and Pearson's Chi-square test of independence with a *P* value ≤ 0.05 considered statistically significant. An ethical clearance was taken from the school of Pharmacy, University of Gondar, and all respondents were asked for their consent before participation in the study.

## 3. Result

Forty-seven pharmacists working in the community pharmacies located in Gondar town completed and returned the questionnaires making a 100% response rate. The majority were male (*N* = 32, 68.1%), in the 23–28-year age group (*N* = 26, 55.3%), at least B. Pharm degree holders (*N* = 25, 53.2%), with work experience of 4 years and below (*N* = 25, 53.2%), and employee (*N* = 28, 59.6%). Thirty-four (72.3%) respondents work 6 days and above per week. An increase in frequency of risks and actual dispensing errors was opined by 21 (44.7%) and 20 (42.6%) respondents, respectively (Table 1).

Perceived factors that contribute to dispensing errors in community pharmacies were assessed using five-point Likert-type questions as shown in Table 2. When the factors are placed in descending order as usual causes of dispensing errors (sometimes/often/very often), poor prescription handwriting (*N* = 45, 95.8%) and similar/confusing names (*N* = 35, 74.4%) are on the top while job dissatisfaction (*N* = 17, 36.2%) is on the bottom.

Similarly, all the listed strategies were taken as very effective measures (sometimes/often/very often) to reduce dispensing errors by 41 (87.2%) to 45 (95.7%) respondents (Table 3).

Perceived dispensing errors commonly happening in community pharmacies (sometimes/often/very often) were dispensing contraindicated drugs (*N* = 29, 61.8%), dispensing with wrong dosing instruction (*N* = 24, 51.1%), dispensing wrong type of drug (*N* = 21, 44.7%), and dispensing wrong dosage form (*N* = 19, 40.4%) (Table 4).

Pearson's Chi-square test of independence demonstrated the presence of association between sex (male/female) of respondents and the opinion that there is an increase in frequency of actual dispensing errors in community pharmacies. Thus, males were more likely to suggest that there is an increase in actual dispensing errors compared to females (17 (53.1%) versus 3 (20%); *P* = 0.032; Table 5).

Mann*–*Whitney *U* test was carried out to test group differences (based on demographic factors) on the perceived factors contributing to dispensing errors and strategies to minimize dispensing errors as well as common dispensing errors happening at the level of community pharmacies in Gondar town, Northwest Ethiopia. There were differences regarding some of the factors as causes for dispensing errors based on age (one factor), educational status (four factors), and work experience (one factor). Age (*P* = 0.032), educational level (*P* = 0.006), and work experience (*P* = 0.015) affected respondents' opinion on poor prescription handwriting as a cause for dispensing errors. Based on educational level, there were differences in considering interruption (*P* = 0.023), design of dispensary (*P* = 0.039), and lack of privacy (*P* = 0.043) as factors contributing to dispensing errors (Table 6).

Similarly, Mann*–*Whitney *U* test on perceived strategies that can minimize dispensing errors revealed age, sex, work experience, and dispensing frequency based differences. Group differences were noted in relation to improving prescription handwriting based on sex (*P* = 0.007), age (*P* = 0.009^*∗*^), and work experience (*P* = 0.030). In addition, the following differences were also found: reducing workloads on pharmacist versus age (*P* = 0.096); keeping drug knowledge up-to-date versus sex (*P* = 0.003) and additional work experience (*P* = 0.034); having drug names that are distinctive versus dispensing frequency (*P* = 0.028); systematic dispensing workflow versus age (*P* = 0.044); having mechanism for checking dispensing procedures versus age (*P* = 0.049) (Table 7).

In relation to perceived type of common dispensing errors, group differences were observed only on dispensing with wrong dosing instruction. Thus, there were age (*P* = 0.027), work experience (*P* = 0.004), and pharmacy ownership (*P* = 0.008) based opinion differences (Table 8).

## 4. Discussion

Dispensing medication is inherently risky and dispensing errors are inevitable occurrences in community pharmacies across the world [9]. This is the first study to evaluate the attitude towards dispensing error of pharmacists working in the private setting in Ethiopia. The settings were chosen because they are the most easily accessible facilities to the community and there is high patient flow and dispensing practice. Nearly half of the respondents agreed that the frequency of risks as well as actual dispensing errors is increasing and there is no significant difference (*P* > 0.05) among participants on these views except for sex (*P* = 0.032). Male pharmacists were more likely to suggest that there is an increase in actual dispensing errors compared to females. Despite this, the finding signals the need for further prospective studies on estimating actual dispensing errors and implementing strategies to minimize dispensing errors. In our study, 20 (42.6%) respondents opined that actual dispensing errors are increasing and this is somehow lower compared to 55.5% and 47% respondents in previous studies conducted in Saudi Arabia and Australia [14, 15].

All the listed factors were well recognized by the respondents as a potential cause for dispensing errors. Poor prescription writing is cited as the most frequent cause of dispensing errors followed by confusing drug names. Both factors were reported in previous studies as major causes of dispensing errors [15, 16]. Respondents who are in the age group of 29–51 years, degree holders, and with more experience rated highly (*P* < 0.05) poor prescription handwriting when compared to their counter groups. It seems that the ability to detect dispensing error increases with age, educational status, and work experience of pharmacists. Despite the fact that there is a need to improve job satisfaction among pharmacists working in Ethiopia [17], it is the factor least appreciated by the respondents as a cause for dispensing errors. Most of the other factors were acknowledged relatively similarly and no significant group differences were found. The exceptions were on the effects of interruption, design of dispensary, and lack of privacy. Respondents with higher educational status rate interruption, design of dispensary, and lack of privacy as factors contributing to dispensing errors highly (*P* < 0.05) than diploma holders. It seems that, in private pharmacy establishments where there is high patient flow and absence of pharmacy assistants, the above factors are meaningful causes of dispensing errors. A similar finding is reported by Al-Arifi [15].

All the strategies listed in the present study were strongly appreciated by the respondents for reducing dispensing errors and this is consistent with previous studies [14, 15]. Labeling and storage of containers in the dispensary, interruptions, and distractions were also identified as main causes of dispensing errors in a hospital setting [18]. However, group differences based on age, sex, work experience, and dispensing frequency were found. Most of the differences in opinions were based on age and thus the older age group were more likely to rate the strategies highly (*P* < 0.05) than their counter groups. Since in our study age is associated with work experience (*P* < 0.05), thus the older group seems to have more experience to appreciate the strategies highly than their counter groups.

Several types of dispensing errors were acknowledged by the respondents but dispensing contraindicated drugs and dispensing with wrong dosing instructions were found to be on top of the others. It seems that even with the routine dispensing practice characterized by provision of dosing instruction [4], there exists gap making interventions mandatory. The older aged group, more experienced ones, and the owners rated dispensing with wrong dosing instruction significantly higher (*P* < 0.05) than their counter groups.

This study however has limitation of small sample size. This is because the number of pharmacists engaged in community pharmacy setting is low in the town. Thus, it may not be generalized to community pharmacists in the nation.

The findings of this study imply that community pharmacists believe that the risks and actual dispensing errors are increasing in the area. Perceived factors causing dispensing errors were related to prescribing quality and design of dispensary as well as dispensing procedures. Thus, training to minimize dispensing errors is very much needed and should also consider the identified factors.

## 5. Conclusion

The risks and actual dispensing errors are increasing in community pharmacies according to the respondents and several contributing factors and strategies against dispensing errors were identified. Most of the time when there are group differences, it is either because of educational level or age of the participants. We suggest further prospective studies in estimating the actual dispensing errors and interventions shall be inclusive of the strategies identified in the present study.

## Figures and Tables

**Table 1:** Demographic characteristics and additional responses (*N* = 47).

Variables	*N* (%)
Sex	
Female	15 (31.9)
Male	32 (68.1)
Age in year (mean = 30.6, SD = 6.9)	
23–28	26 (55.3)
29–51	21 (44.7)
Educational level	
Diploma	22 (46.8)
BPharm	24 (51.1)
MSc	1 (2.1)
Work experience in community pharmacy (year) (mean = 5.2, SD = 3.6)	
1–4 year	25 (53.2)
5–16 year	22 (46.8)
Additional work experience	
Yes	23 (48.9)
No	24 (51.1)
Pharmacy ownership	
Owner	19 (40.4)
Employee	28 (59.6)
Frequency of participating in dispensing within a week	
≤5 days/week	13 (27.7)
≥6 days/week	34 (72.3)
Opinions on whether the risk of dispensing errors is increasing.	
No	26 (55.3)
Yes	21 (44.7)
Opinions whether the actual errors in dispensing are becoming more common	
No	27 (57.4)
Yes	20 (42.6)

**Table 2:** Perceived factors contributing to the dispensing errors (*N* = 47).

Variables	Responses *N* (%)				
	Never	Rarely	Sometimes	Often	Very often
Poor prescription hand writing	0	2 (4.2)	10 (21.3)	13 (27.7)	22 (46.8)
Similar/confusing names	1 (2.1)	11 (23.5)	18 (38.3)	16 (34.0)	1 (2.1)
Work load	3 (6.4)	16 (34.0)	12 (25.5)	9 (19.2)	7 (14.9)
Lack of time to talk with patients	9 (19.2)	12 (25.5)	17 (36.2)	5 (10.6)	4 (8.5)
Packaging & labeling	7 (14.9)	15 (31.9)	19 (40.4)	4 (8.5)	2 (4.3)
Interruption	5 (10.6)	17 (36.2)	17 (36.2)	3 (6.4)	5 (10.6)
Design of dispensary	10 (21.3)	12 (25.5)	12 (25.5)	7 (14.9)	6 (12.8)
Pharmacist fatigue of any cause	3 (6.4)	21(44.7)	14 (29.8)	7 (14.9)	2 (4.2)
Noise	10 (21.3)	14 (29.8)	12 (25.5)	5 (10.6)	6 (12.8)
Lack of privacy	14 (29.8)	10 (21.3)	9 (19.1 )	4 (8.5)	10 (21.3)
Job dissatisfaction	14 (29.8)	16 (34.0)	8 (17.0)	4 (8.5)	5 (10.7)

*Note*. The above responses were arranged in descending order for the sum of responses (sometimes/often/very often).

**Table 3:** Perceived strategies that may reduce the risk of dispensing errors (*N* = 47).

Variables	Responses *N* (%)				
	Never	Rarely	Sometimes	Often	Very often
Improving prescription hand writing	0	2 (4.2)	8 (17.0)	7 (14.9)	30 (63.9)
Checking original prescription	0	2 (4.2)	6 (12.8)	13 (27.7)	26 (55.3)
Having mechanism for checking dispensing procedures	0	2 (4.2)	7 (14.9)	17 (36.2)	21 (44.7)
Counseling patients at the time of supply	1 (2.1)	1 (2.1)	3 (6.4)	10 (21.3)	32 (68.1)
Keeping drug knowledge up-to-date	1 (2.1)	2 (4.2)	6 (12.8)	7 (14.9)	31 (66.0)
Systematic dispensing workflow	0	3 (6.4)	13 (27.7)	15 (31.9)	16 (34.0)
Privacy when counseling patients	1 (2.1)	3 (6.4)	4 (8.5)	10 (21.3)	29 (61.7)
Having drug names that are distinctive	1 (2.1)	4 (8.5)	10 (21.3)	18 (38.3)	14 (29.8)
Reducing workloads on pharmacist	1 (2.1)	5 (10.7)	10 (21.3)	15 (31.9)	16 (34.0)
Improving packaging & labeling	2 (4.2)	4 (8.5)	10 (21.3)	17 (36.2)	14 (29.8)

*Note*. The above responses were arranged in descending order for the sum of responses (sometimes/often/very often).

**Table 4:** Perceived type of dispensing errors in community pharmacies (*N* = 47).

Variables	Responses *N* (%)				
	Never	Rarely	Sometimes	Often	Very often
Dispensing contraindicated drug	9 (19.1)	9 (19.1)	20 (42.7)	9 (19.1)	0
Dispensing with wrong dosing instruction	5 (10.6)	18 (38.3)	14 (29.9)	9 (19.1)	1 (2.1)
Dispensing wrong type of drug	11 (23.4)	15 (31.9)	14 (29.8)	5 (10.6)	2 (4.3)
Dispensing wrong dosage form	11 (23.4)	17 (36.2)	12 (25.5)	5 (10.6)	2 (4.3)

*Note*. The above responses were arranged in descending order for the sum of responses (sometimes/often/very often).

**Table 5:** Relationship between demographic factors and opinion if frequency of risk and actual dispensing errors are increasing (*N* = 47).

Variables	Responses *N* (%)			
	Risk of dispensing errors is increasing (*N* = 21)		Actual dispensing errors are increasing (*N* = 20)	
	*N* (%)		*N* (%)	
Sex				
Female (*N* = 15)	4 (26.7%)	*X* ^2^ = 2.892, df = 1, *P* = 0.089	3 (20%)	*X* ^2^ = 4.584, df = 1, *P* = 0.032^*∗*^
Male (*N* = 32)	17 (53.1%)		17 (53.1%)	
Age (years)				
23–28 (*N* = 26)	9 (34.6%)	*X* ^2^ = 2.385, df = 1, *P* = 0.122	10 (38.5%)	*X* ^2^ = 0.399, df = 1, *P* = 0.528
29–51 (*N* = 21)	12 (57.1%)		10 (47.6%)	
Educational level				
Diploma (*N* = 22)	8 (36.4%)	*X* ^2^ = 1.158, df = 1, *P* = 0.282	9 (40.9%)	*X* ^2^ = 0.046, df = 1, *P* = 0.831
BPharm degree and above (*N* = 25)	13 (52%)		11 (44%)	
Work experience (years)				
1–4 year (*N* = 25)	10 (40%)	*X* ^2^ = 0.473, df = 1, *P* = 0.491	11 (44%)	*X* ^2^ = 0.046, df = 1, *P* = 0.831
5–16 year (*N* = 22)	11 (50%)		9 (40.9%)	
Additional work experience				
No (*N* = 24)	11 (45.8%)	*X* ^2^ = 0.026, df = 1, *P* = 0.871	9 (37.5%)	*X* ^2^ = 0.512, df = 1, *P* = 0.474
Yes (*N* = 23)	10 (43.5%)		11 (47.8%)	
Pharmacy ownership				
Owner (*N* = 19)	8 (42.1%)	*X* ^2^ = 0.086, df = 1, *P* = 0.770	6 (31.6%)	*X* ^2^ = 1.571, df = 1, *P* = 0.210
Employee (*N* = 28)	13 (46.4%)		14 (50%)	
Dispensing practice				
≤5 days/week (*N* = 13)	4 (30.8%)	*X* ^2^ = 1.407, df = 1, *P* = 0.236	5 (38.5%)	*X* ^2^ = 1.230, df = 1, *P* = 0.726
≥6 days/week (*N* = 34)	17 (50%)		15 (44.1%)	

*Note*. ^*∗*^= significant (*P* ≤ 0.05), df = degrees of freedom, *X*^2^ = Pearson's Chi-square value.

**Table 6:** Perceived factors contributing to the dispensing errors (*N* = 47).

Factors for dispensing errors	Independent variables						
	Sex	Age	Educational level	Work experience	Additional work experience	Pharmacy ownership	Dispensing frequency
Poor prescription hand writing	*P* = 0.166	*P* = 0.032^*∗*^	*P* = 0.006^*∗*^	*P* = 0.015^*∗*^	*P* = 0.545	*P* = 0.429	*P* = 0.451
Similar/confusing names	*P* = 0.781	*P* = 0.089	*P* = 0.644	*P* = 0.326	*P* = 0.528	*P* = 0.070	*P* = 0.308
Packaging & labeling	*P* = 0.366	*P* = 0.813	*P* = 0.113	*P* = 0.290	*P* = 0.121	*P* = 0.067	*P* = 0.960
Pharmacist fatigue of any cause	*P* = 0.560	*P* = 0.452	*P* = 0.785	*P* = 0.829	*P* = 0.821	*P* = 0.652	*P* = 0.205
Job dissatisfaction	*P* = 0.972	*P* = 0.386	*P* = 0.370	*P* = 0.666	*P* = 0.389	*P* = 0.063	*P* = 0.236
Work load	*P* = 0.333	*P* = 0.543	*P* = 0.529	*P* = 0.791	*P* = 0.792	*P* = 0.189	*P* = 0.242
Noise	*P* = 0.354	*P* = 0.758	*P* = 0.268	*P* = 0.653	*P* = 0.810	*P* = 0.200	*P* = 0.174
Interruption	*P* = 0.280	*P* = 0.143	*P* = 0.023^*∗*^	*P* = 0.515	*P* = 0.754	*P* = 0.032	*P* = 0.395
Design of dispensary	*P* = 0.575	*P* = 0.792	*P* = 0.039^*∗*^	*P* = 0.388	*P* = 0.639	*P* = 0.876	*P* = 0.503
Lack of privacy	*P* = 0.606	*P* = 0.429	*P* = 0.043^*∗*^	*P* = 0.630	*P* = 0.444	*P* = 0.429	*P* = 0.427
Lack of time to talk with patients	*P* = 0.053	*P* = 0.345	*P* = 0.472	*P* = 0.188	*P* = 0.515	*P* = 0.964	*P* = 0.872

*Note*. 5 point likert scale (0 = never, 4 = very often), ^*∗*^= statistically significant at *P* ≤ 0.05, Mann-Whitney *U* test.

**Table 7:** Perceived solutions for minimizing dispensing errors (*N* = 47).

Perceived solutions for minimizing dispensing errors	Independent variables						
	Sex	Age	Educational level	Work experience	Additional work experience	Pharmacy ownership	Dispensing frequency
Improving prescription hand writing	*P* = 0.007	*P* = 0.009^*∗*^	*P* = 0.217	*P* = 0.030^*∗*^	*P* = 0.518	*P* = 0.820	*P*= 0.397
Reducing workloads on pharmacist	*P* = 0.253	*P* = 0.096^*∗*^	*P* = 0.243	*P* = 0.139	*P* = 0.633	*P* = 0.325	*P* = 0.519
Keeping drug knowledge up-to-date	*P* = 0.003	*P* = 0.127	*P* = 0.382	*P* = 0.119	*P* = 0.034^*∗*^	*P* = 0.511	*P* = 0.323
Having drug names that are distinctive	*P* = 0.684	*P* = 0.167	*P* = 0.206	*P* = 0.576	*P* = 0.294	*P* = 0.802	*P* = 0.028^*∗*^
Improving packaging & labeling	*P* = 0.504	*P* = 0.169	*P* = 0.247	*P* = 0.456	*P* = 0.903	*P* = 0.359	*P* = 0.089
Checking original prescription	*P* = 0.849	*P* = 0.225	*P* = 0.434	*P* = 0.758	*P* = 0.906	*P* = 0.971	*P* = 0.552
Systematic dispensing workflow	*P* = 0.165	*P* = 0.044^*∗*^	*P* = 0.158	*P* = 0.389	*P* = 0.841	*P* = 0.794	*P* = 0.042
Having mechanism for checking dispensing procedures	*P* = 0.597	*P* = 0.049^*∗*^	*P* = 0.774	*P* = 0.135	*P* = 0.630	*P* = 0.815	*P* = 0.465
Counseling patients at the time of supply	*P* = 0.291	*P* = 0.515	*P* = 0.866	*P* = 0.640	*P* = 0.492	*P* = 0.262	*P* = 0.385
Privacy when counseling patients	*P* = 0.555	*P* = 0.180	*P* = 0.404	*P* = 0.027^*∗*^	*P* = 0.695	*P* = 0.318	*P* = 0.468

*Note*. 5 point likert scale (0 = never, 4 = very often), ^*∗*^= statistically significant at *P* ≤ 0.05, Mann-Whitney *U* test.

**Table 8:** Perceived type of common dispensing errors in community pharmacies (*N* = 47).

Perceived type of common dispensing errors	Independent variables						
	Sex	Age	Educational level	Work experience	Additional work experience	Pharmacy ownership	Dispensing frequency
Dispensing wrong type of drug	*P* = 0.250	*P* = 0.158	*P* = 0.358	*P* = 0.106	*P* = 0.667	*P* = 0.339	*P* = 0.951
Dispensing with wrong dosing instruction	*P* = 0.382	*P* = 0.027^*∗*^	*P* = 0.110	*P* = 0.004^*∗*^	*P* = 0.250	*P* = 0.008^*∗*^	*P* = 0.337
Dispensing contraindicated drug	*P* = 0.524	*P* = 0.285	*P* = 0.928	*P* = 0.606	*P* = 0.509	*P* = 0.846	*P* = 0.950
Dispensing wrong dosage form	*P* = 0.269	*P* = 0.229	*P* = 0.549	*P* = 0.329	*P* = 0.642	*P* = 0.668	*P* = 0.710

*Note*. 5 point likert scale (0 = never, 4 = very often), ^*∗*^= statistically significant at *p* ≤ 0.05, Mann-Whitney *U* test.

## Additional Points

*Availability of Data and Material.* The main document from which this manuscript was developed is deposited in the Pharmacy School of University of Gondar.

## References

[1] Adepu R., Nagavi B. (2006). General practitioners' perceptions about the extended roles of the community pharmacists in the state of Karnataka: a study. *Indian Journal of Pharmaceutical Sciences*.

[2] WHO. (1987). *The Rational Use of Drugs. Report of a Conference of Experts, Nairobi, 25–29 November 1985*.

[3] International Pharmaceutical Federation Standards for quality of pharmacy services.

[4] Ax F., Brånstad J.-O., Westerlund T. (2010). Pharmacy counselling models: a means to improve drug use. *Journal of Clinical Pharmacy and Therapeutics*.

[5] Mishra P., Hansen E. H., Sabroe S., Kafle K. K. (2006). Adherence is associated with the quality of professional-patient interaction in Directly Observed Treatment Short-course, DOTS. *Patient Education and Counseling*.

[6] US Food Medication Errors, US Department of Health and Human Services. http://www.fda.gov/Drugs/DrugSafety/MedicationErrors/default.htm.

[7] Szeinbach S., Seoane-Vazquez E., Parekh A., Herderick M. (2007). Dispensing errors in community pharmacy: perceived influence of sociotechnical factors. *International Journal for Quality in Health Care*.

[8] Cheung K.-C., Bouvy M. L., De Smet P. A. G. M. (2009). Medication errors: the importance of safe dispensing. *British Journal of Clinical Pharmacology*.

[9] James K. L., Barlow D., McArtney R., Hiom S., Roberts D., Whittlesea C. (2009). Incidence, type and causes of dispensing errors: a review of the literature. *International Journal of Pharmacy Practice*.

[10] Puspitasari H. P., Aslani P., Krass I. (2009). How do Australian metropolitan and rural pharmacists counsel consumers with prescriptions?. *Pharmacy World and Science*.

[11] Sada O., Melkie A., Shibeshi W. (2015). Medication prescribing errors in the medical intensive care unit of Tikur Anbessa Specialized Hospital, Addis Ababa, Ethiopia. *BMC Research Notes*.

[12] Feleke S. A., Mulatu M. A., Yesmaw Y. S. (2015). Medication administration error: magnitude and associated factors among nurses in Ethiopia. *BMC Nursing*.

[13] Dedefo M. G., Mitike A. H., Angamo M. T. (2016). Incidence and determinants of medication errors and adverse drug events among hospitalized children in West Ethiopia. *BMC Pediatrics*.

[14] Peterson G. M., Wu M. S. H., Bergin J. K. (1999). Pharmacists' attitudes towards dispensing errors: their causes and prevention. *Journal of Clinical Pharmacy and Therapeutics*.

[15] Al-Arifi M. N. (2014). Community pharmacists' attitudes toward dispensing errors at community pharmacy setting in Central Saudi Arabia. *Saudi Pharmaceutical Journal*.

[16] Knudsen P., Herborg H., Mortensen A. R., Knudsen M., Hellebek A. (2007). Preventing medication errors in community pharmacy: root-cause analysis of transcription errors. *Quality and Safety in Health Care*.

[17] Gebretekle G. B., Fenta T. G. (2013). Assessment of the pharmacist’s workforce in Ethiopia. *Ethiopian Journal of Health Development*.

[18] Beso A., Franklin B. D., Barber N. (2005). The frequency and potential causes of dispensing errors in a hospital pharmacy. *Pharmacy World and Science*.

